# Extended-spectrum beta-lactamase (ESBL)-producing *Escherichia coli* and *Acinetobacter baumannii* among horses entering a veterinary teaching hospital: The contemporary "Trojan Horse"

**DOI:** 10.1371/journal.pone.0191873

**Published:** 2018-01-30

**Authors:** Birgit Walther, Katja-Sophia Klein, Ann-Kristin Barton, Torsten Semmler, Charlotte Huber, Silver Anthony Wolf, Karsten Tedin, Roswitha Merle, Franziska Mitrach, Sebastian Guenther, Antina Lübke-Becker, Heidrun Gehlen

**Affiliations:** 1 Institute of Microbiology and Epizootics, Freie Universität Berlin, Berlin, Germany; 2 Advanced Light and Electron Microscopy, Robert Koch Institute, Berlin, Germany; 3 Equine Clinic, Surgery and Radiology, Freie Universität Berlin, Berlin, Germany; 4 Microbial Genomics (NG1), Robert Koch Institute, Berlin, Germany; 5 Institute for Veterinary Epidemiology and Biostatistics, Freie Universität Berlin, Berlin, Germany; 6 Institute of Biotechnology, Faculty of Environment and Natural Sciences, Brandenburg University of Technology Cottbus-Senftenberg, Senftenberg, Germany; 7 Institute of Animal and Environmental Hygiene, Freie Universität Berlin, Berlin Germany; 8 Institute of Pharmacy, Pharmaceutical Biology, Ernst Moritz Arndt Universität Greifswald, Greifswald Germany; Ross University School of Veterinary Medicine, SAINT KITTS AND NEVIS

## Abstract

Pathogens frequently associated with multi-drug resistant (MDR) phenotypes, including extended-spectrum beta-lactamase (ESBL)-producing Enterobacteriaceae (ESBL-E) and *Acinetobacter baumannii* isolated from horses admitted to horse clinics, pose a risk for animal patients and personnel in horse clinics. To estimate current rates of colonization, a total of 341 equine patients were screened for carriage of zoonotic indicator pathogens at hospital admission. Horses showing clinical signs associated with colic (n = 233) or open wounds (n = 108) were selected for microbiological examination of nostril swabs, faecal samples and wound swabs taken from the open wound group. The results showed alarming carriage rates of Gram-negative MDR pathogens in equine patients: 10.7% (34 of 318) of validated faecal specimens were positive for ESBL-E (94%: ESBL-producing *Escherichia coli*), with recorded rates of 10.5% for the colic and 11% for the open wound group. 92.7% of the ESBL-producing *E*. *coli* were phenotypically resistant to three or more classes of antimicrobials. *A*. *baumannii* was rarely detected (0.9%), and all faecal samples investigated were negative for *Salmonella*, both directly and after two enrichment steps. Screening results for the equine nostril swabs showed detection rates for ESBL-E of 3.4% among colic patients and 0.9% in the open wound group, with an average rate of 2.6% (9/340) for both indications. For all 41 ESBL-producing *E*. *coli* isolated, a broad heterogeneity was revealed using pulsed-field gel electrophoresis (PFGE) patterns and whole genome sequencing (WGS) -analysis. However, a predominance of sequence type complex (STC)10 and STC1250 was observed, including several novel STs. The most common genes associated with ESBL-production were identified as *bla*_CTX-M-1_ (31/41; 75.6%) and *bla*_SHV-12_ (24.4%).

The results of this study reveal a disturbingly large fraction of multi-drug resistant and ESBL-producing *E*. *coli* among equine patients, posing a clear threat to established hygiene management systems and work-place safety of veterinary staff in horse clinics.

## Introduction

Multi-drug resistant (MDR) pathogens, especially extended-spectrum beta-lactamase (ESBL)-producing Enterobacteriaceae, have frequently been reported as a cause of severe infections in horses, which has become an issue of increasing importance in veterinary infection control and biosafety in equine clinical settings [1–6]. While there is no significant clinical difference for individual horses with respect to the particular pathogen source in case of infection, the distinction between an endogenous self-infection versus an exogenous cross-infection is crucial from the standpoint of epidemiology and disease prevention (http://www.who.int/water_sanitation_health). Thus, precise information on the local endemic load and epidemiology of MDR carried by patients entering a hospital is crucial for infection control policies [7]. Such data can be used for the targeted development of prevention strategies to protect the horse from exogenous as well as endogenous infection sources [3]. In the past, severe infections with Gram-negative bacteria in hospitalized horses have generally been reported in association with *Salmonella* infections [8–10] and ESBL-producing Enterobacteriaceae [3, 11]. Moreover, equine patients colonized or infected with *Acinetobacter baumannii* [12, 13] might pose a risk for themselves or other patients within a clinical environment. Consequently, the aim of this study was to determine the carriage rate of these pathogens which harbor the potential for nosocomial and zoonotic transmission in equine clinical settings as a first step for subsequent implementation of more specific hygiene barriers. Accordingly, ESBL-producing Enterobacteriaceae, *Salmonella* and *A*. *baumannii* were selected as indicator pathogens to estimate the influx of Gram-negative MDR strains.

## Material and methods

### Participants, setting and sample collection

As part of a hygiene intervention program, a prospective prevalence study was conducted at the clinic for horses at the Freie Universität Berlin with sampling taking place over a two-year period, from April to October in 2014 and again in 2015. Horses showing clinical signs associated with either “colic” or an “open wound” at hospital admission were included. Inclusion criteria for patient samples were as follows: sterile cotton swabs with Amies transport medium (Mast Diagnostica, Reinfeld) of both anterior nostrils taken on arrival without delay and faecal samples of the first defecation after admission within 120 min. Horses leaving the clinic within 24h after admission were excluded from the study groups. Those horses which did not survive 24h due to euthanasia or sudden death were included, since these animals were regarded as fully hospitalized.

In addition, a wound swab was taken immediately upon admission from the “open wound” patients within the reception area. One person (K-S.K.) was trained to take the microbiological samples and collected the data on horse patients during both study periods.

### Ethics

According to the German regulations for research with animal subjects, taking swabs from anterior nasal cavity of equine patients at hospital admission in the context of a hygiene evaluation does not require approval (Landesamt für Gesundheit und Soziales, Berlin, 14.07.2014).

### Phenotypic screening procedures applied for distinct detection of *A*. *baumannii*, ESBL-producing Enterobacteriaceae and *Salmonella* species

Faecal specimens, nostril- and wound swabs were initially cultured on CHROMagar™ Acinetobacter (Mast Diagnostica, Germany). ChromID (bioMérieux, Germany) supplemented with 4mg/l cefotaxime as described previously [14]. For *Salmonella* screening, 10g of each fecal sample was enriched overnight in *Salmonella* Rappaport-Vassiliadis enrichment broth (Oxoid, Germany) at 42°C. For further cultivation, Gassner Agar (Oxoid, Germany) and XLD Agar (Becton Dickinson diagnostics, Germany) was used. A second enrichment step using a 1:100 dilution of the initial broth was also performed. Identification of indicator species was determined using MALDI-TOF MS (Bruker, Germany) and the Vitek2 GN_ID_card (bioMérieux, Germany). All Enterobacteriaceae showing initial growth on chromogenic screening plates were tested for ESBL-production using the phenotypic confirmatory test, performed and interpreted according to CLSI standards [15]. Colonies showing differences in phenotypical appearance per bacterial species were included in further sub-typing procedures.

Minimal inhibitory concentration (MIC)-testing was carried out using the VITEK®2 system (BioMérieux, Germany) including ampicillin, amoxicillin-clavulanic acid piperacillin, cephalexin, cefpodoxim, cephalexin, amikacin, gentamicin, enrofloxacin, marbofloxacin, tetracycline, nitrofurantoin, chloramphenicol, and trimethoprim-sulfamethoxazole according to the standards described in the CLSI documents M31-A3 and M100-S21 [16, 17], and for Ceftiofur MIC interpretation in *Acinetobacter baumannii* Klotz *et al*. 2017 [18].

### Statistical analysis

Statistical analyses were carried out using SPPS version 24 (IBM). In order to investigate the influence of clinical background/admission at the horse clinic (clinical signs of colic or having an open wound) and seasonal variation on the occurrence of Enterobacteriaceae-ESBL, a logistic regression model was adapted. P-values < 0.05 were regarded statistically significant.

### Molecular characterization of ESBL-producing *E*. *coli*

Molecular typing for ESBL-producing *E*. *coli* included genomic macro-restriction using *Xba*I was followed by PFGE and pattern analysis using Bionumerics [3, 19]. All confirmed ESBL-producing *E*. *coli* isolates were whole-genome sequenced (WGS) using Illumina MiSeq 300 bp paired-end sequencing with an obtained coverage > 90x. After quality control using the NGS tool kit13 (70% of bases with a phred score >20), high-quality filtered reads were used for *de novo* assembly into contiguous sequences (contigs) and subsequently into scaffolds using SPAdes v3.9. Assembled draft genomes of the isolates were annotated using the rapid annotation server RAST. WGS data were used for genotypic characterization including the determination of the sequence type (ST) (MLSTFinder [20]), transferable resistance genes (ResFinder 2.1 [21], threshold: 98% ID, 90% minimum length) and plasmids (PlasmidFinder 1.3, [22] threshold: 95% ID).

In order to compare the genomes at high resolution, we used the maximum common genome (MCG) that is defined by those orthologous genes present in all genomes [23]. To obtain this, we clustered the coding sequences based on the parameters of sequence similarity (min. 70%) and coverage (min. 90%) and defined those genes that were present in each genome while fulfilling the threshold parameters as MCG which in this case comprised 2,876 genes. Next, the allelic variants of these genes were extracted from all genomes by a blast-based approach, then aligned individually for each gene and concatenated, resulting in an alignment of 2.520 Mbp for these strains. This alignment was used to generate a maximum likelihood phylogenetic tree with RAxML 8.1 [24].

## Results

Overall, 341 equine patients were enrolled in this study, 174 horses in 2014 and 167 in 2015, respectively. The average duration of hospital stay was 7,8 (median: 4) days for all equine patients included in the study. Twenty-five horses admitted with clinical signs of colic died or were euthanized within the first 24h hours. Since they did not leave the hospital premises within that time, they were judged as horses with one day hospital stay. With this constraint, the average duration of hospital stay recorded for the colic group was 5,3 (median: 4) days and 13,2 (median: 10) for the open wound group.

Only those specimens which met the inclusion criteria were considered for further analysis. Consequently, a single nostril swab and 23 faecal samples were either excluded due to an exceeded time frame or were not available. For the faecal samples, missing samples were largely due to the lack of defecation within the predetermined post-admission time allowed for sampling (2 h).

For the 340 valid nostril swabs, 2.6% were found positive for ESBL-E (1.4% *E*. *coli*, 1.4% *Enterobacter cloacae*, *0*.*3% Citrobacter freundii*, 0.3% *Leclercia sp*.) and 1.5% for *A*. *baumannii*.

A total of 318 faecal samples meeting the sampling criteria were obtained, for which an isolation rate of 10.7% ESBL-E (10.1% *E*. *coli*, 0.9% *Citrobacter freundii*, 0.6% *E*. *cloacae* and 0.3% *Klebsiella pneumoniae*) and 1.4% *A*. *baumannii*. No *Salmonella*-positive samples were identified in period of this study.

**Fig 1:**
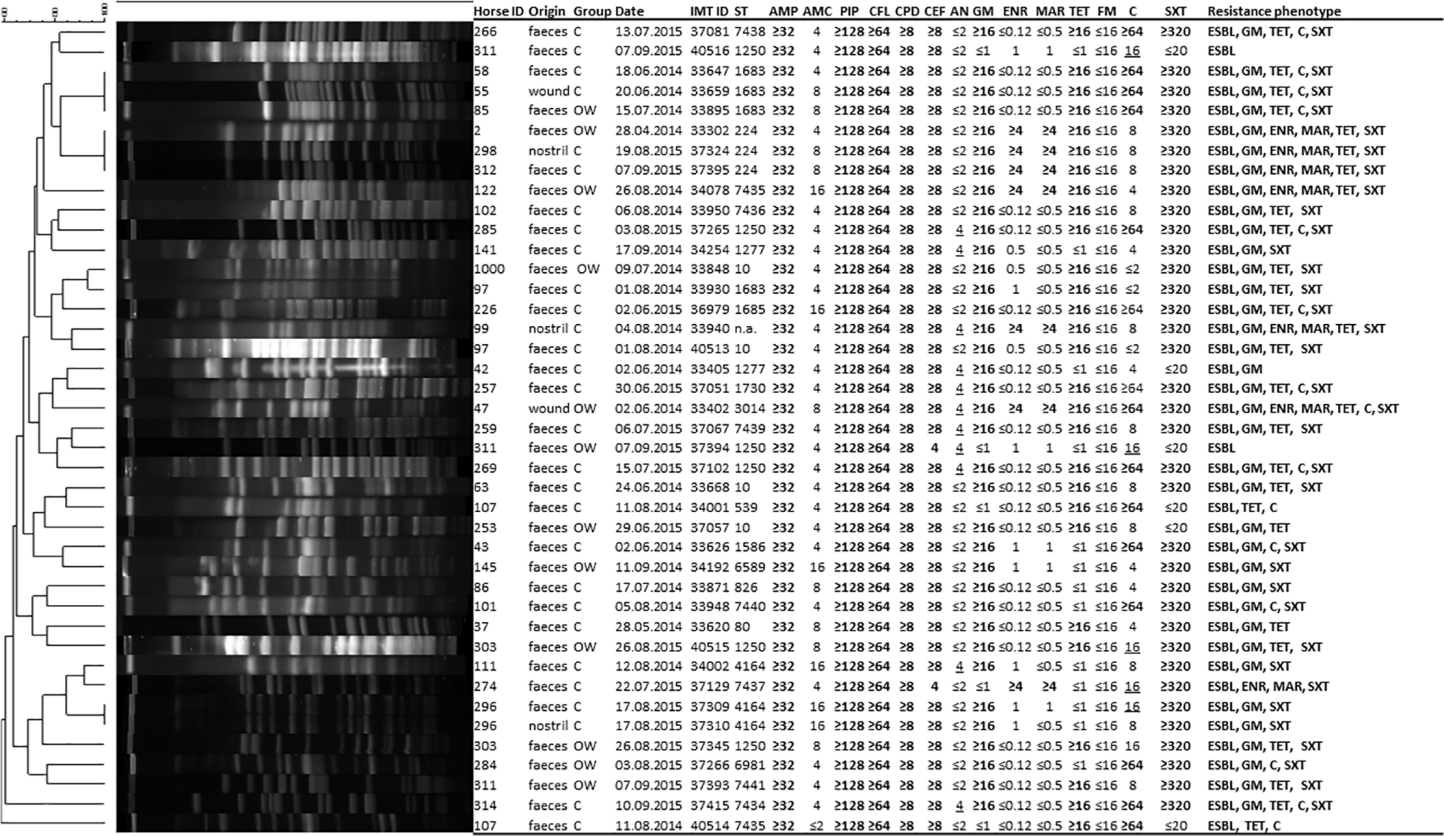
PFGE-analysis of ESBL-producing *E*. *coli* isolated from specimens obtained from horses at hospital admission. Dendrogram (percent similarity) showing DNA restriction pattern after digestion with XbaI for 41 *E*. *coli* isolated from horses directly at hospital admission. PFGE analysis by use of bionumerics® (unweighted-pair group method using average linkages), dice coefficient, 1.5% tolerance and 0.5% optimization indicated limited clonal relatedness. Antimicrobial susceptibility testing results VITEK®2 system (BioMérieux, Germany) for all ESBL-producing isolates revealed that multi-drug resistance is common. Plain numbers = susceptible-, bold = resistant-, underlined = intermediate phenotype. Abbreviations: Horse ID, individual number for each enrolled equine patient; OW, horse of the open wound group; C, horse of the colic group; IMT ID, strain collection number at IMT; ST, sequence type; AMP, ampicillin; AMC, amoxicillin-clavulanic acid; PIP, piperacillin; CFL, cephalexin; CPD, cefpodoxim; CEF, cephalexin; AN, amikacin; GM, gentamicin; ENR, enrofloxacin; MAR, marbofloxacin; TET, tetracycline; FM, nitrofurantoin; C, chloramphenicol; SXT, trimethoprim-sulfamethoxazole.

One ESBL-producing *E*. *coli* was isolated from a wound swab (Fig 1).

The logistic regression model revealed that neither the horse group studied nor seasonal differences had a significant influence on the occurrence of Enterobacteriaceae-ESBL (p-values: overall 0.889, horse group 0.660, screening period 0.866). The regression coefficients showed that horses with open wounds (b = 0.066) as well as horses admitted in the first screening period (b = 0.161) tended to have a higher probability for enteral carriage Enterobacteriaceae-ESBL. Note that as some of the samples collected were positive for more than one ESBL-producing Enterobacteriaceae spp., the isolate number does not necessarily match with the number of positive specimens presented in Table 1.

**Table 1:** Results from Gram-negative indicator pathogen screening of equine patients at hospital admission.

	Screening period I & II						Screening period I						Screening period II					
	n = 341 horses						Apr-Oct 2014 (n = 174 horses)						Apr-Oct 2015 (n = 167 horses)					
Specimens (admission)	total (I+II)		colic		open wound		total (I)		colic		open wound		total (II)		colic		open wound	
	n	%	n	%	n	%	n	%	n	%	n	%	n	%	n	%	n	%
**Nostril swabs (valid)**	340	100	232	100	108	100	173	100	113	100	60	100	167	100	119	100	48	100
**Enterobacteriaceae_ESBL**	9	2.6	8	3.4	1	0.9	4	2.3	3	2.7	1	1.7	5	3.0	5	4.2	0	0
**ESBL: > 1 isolate**	1	0.3	0	0	1	0.9	1	0.6	0	0	1	1.7	0	0	0	0	0	0
***A*. *baumannii***	5	1.5	4	1.7	1	0.9	2	1.2	2	1.8	0	0	3	1.8	2	1.7	1	2.1
**Faeces (valid)**	318	100	220	100	98	100	166	100	109	100	57	100	152	100	111	100	41	100
**Enterobacteriaceae_ESBL**	34	10.7	23	10.5	11	11.2	19	11.4	12	11.0	7	12.3	15	9.9	11	9.9	4	9.8
**ESBL: > 1 isolate**	4	1.3	2	0.9	2	2.0	1	0.6	1	0.9	0	0.0	3	2.0	1	0.9	2	4.9
***A*. *baumannii***	3	0.9	3	1.4	0	0	0	0	0	0	0	0	3	2.0	3	2.7	0	0
**Salmonella**	0	0	0	0	0	0	0	0	0	0	0	0	0	0	0	0	0	0
**Wound swabs**					108	100					60	100					48	100
**Enterobacteriaceae_ESBL**					1	0.9					1	1.7					0	0
**ESBL: > 1 isolate**					0	0					0	0					0	0
***A*. *baumannii***					1	0.9					0	0					1	2.1

Abbreviations: n: number; Enterobacteriaceae_ESBL: extended-spectrum beta-lactamase-producing Enterobacteriaceae sp.; > 1 isolate: specimens positive for more than one ESBL-producing isolate

**Fig 2:**
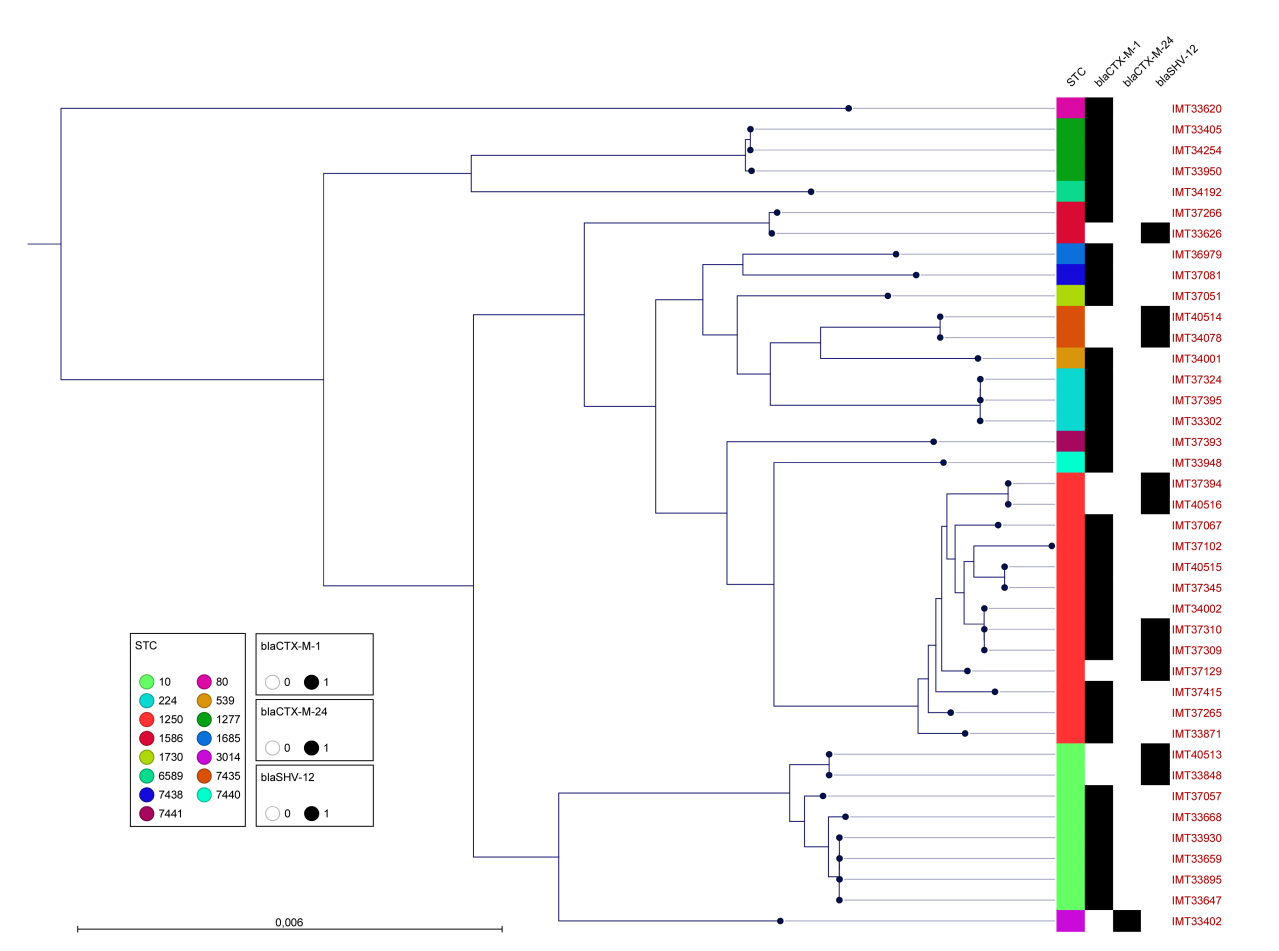
Maximum likelihood tree based on the maximum common genome (MCG). Maximum likelihood tree showing the relatedness of the strains based on their genome sequences together with their corresponding Sequence Type Complex (STC). The occurrences and distribution of *bla*_CTX-M-1,_ *bla*_CTX-M-24_ and *bla*_SHV-12_ in ESBL-producing *E*. *coli* obtained from horses at hospital admission is marked with black squares. While a general broad genomic heterogenicity is obvious, two genetic backgrounds are dominating (STC10 and STC1250).

Comparative PFGE-analysis, AST as well as WGS for MLST assignment and identification of molecular characteristics facilitating ESBL-production was carried out for all 41 *E*. *coli* isolates predominating among the ESBL-producing Enterobacteriaceae spp. Genes associated with ESBL-production detected included *bla*_CTXM-1_ (31/41; 75.6%) and *bla*_SHV-12_ (14.6%) (see Fig 2 for details). Phenotypical resistance in addition to ESBL-production among the 41 *E*. *coli* isolates was notable. Two isolates were ESBL-producers only, one isolate showed resistance towards one further class of antimicrobials, 11 towards two additional classes, 12 towards three classes, 14 towards four classes, and one isolate showed resistance to five additional antibiotic classes (Fig 1 and S1 Table).

PFGE pattern analyses revealed limited clonal relatedness (Fig 2), indicating a broad heterogeneity among the isolates. However, isolates showing indistinguishable PFGE pattern were identified in specimens taken from different body sites, *e*.*g*. from the nasal and feacal samples of horse 296. Moreover, both isolates (IMT37309 and IMT37310) were assigned to ST4164 and were found positive for two different ESBL-gene families (*bla*_CTX-M-1,_ *bla*_SHV-12_). On the other hand, ESBL-producing *E*. *coli* isolated from specimens of different horses from distinct animal owners were found to share a single PFGE pattern, although these horses were admitted to the clinic in different years. Thus, isolates sharing a particular PFGE pattern positive for CTX-M-1 associated with ST224 were likewise obtained from nasal swabs (IMT37324, 08/2015) and from two different faecal samples (IMT37395, 09/2015 and IMT33302, 4/2014) (Fig 2).

MLST revealed 22 different STs, including eight novel STs (ST7434 to ST7441, Fig 2 and Table 1). With regard to genomic lineages, 13 isolates (31.7%) where associated with sequence type complex (STC) 1250 (ST1250, ST826, ST4164, ST7434, ST7437, ST7439), eight (19.5%) with STC 10 (ST10, ST1683) and a further three with ST224 (Figs 1 and 2). A phylogenetic tree generated based on the maximum common genome confirmed the overall broad range of phylogenetic backgrounds (Fig 2), consistent with the PFGE analysis. While the isolates identified as ST1250 and ST10 or single locus variants of those STs formed the two dominating clusters in the genome comparison, the other genomes showed a rather diverse distribution with no similarity to the two clusters or between themselves.

*In silico* plasmid replicon detection revealed a high variety of different types of plasmids including Inc/Rep types IncFIA/B and IncFII, which are commonly found in ESBL-producing *E*. *coli*. One IncFIA plasmid type (pMLST type F-:A8:B-) was present in 15 of the 41 tested *E*. *coli* assigned to 13 different STs, originating from 15 different animals. However, the genetic makeup of resistance genes differed between the strains, arguing against an identical plasmid (S1 Table).

With regard to the nine *A*. *baumannii* isolates detected, only IMT33939 exhibited an MDR phenotype (Table 2). Of note, nostril swab obtained from horse 99 was also positive for ESBL-producing *E*. *coli* (Fig 2).

**Table 2:** Antibiotic susceptibility testing results for *Acinetobacter baumannii* isolated from horses.

Strain ID	AN	CEF*	ENR	GM	IMI	MAR	PIP	TET	TOB	SXT
37247	≤2	**≥8**	≤0.12	≤1	≤1	≤0.5	8	≤1	≤1	≤20
36994	≤2	**≥8**	≤0.12	≤1	≤1	≤0.5	8	≤1	≤1	≤20
36853	≤2	**≥8**	≤0.12	≤1	≤1	≤0.5	8	≤1	≤1	≤20
**33939**	≤2	**≥8**	≤0.12	**≥16**	≤1	≤0.5	8	**≥16**	**8**	**80**
33922	≤2	**≥8**	≤0.12	≤1	≤1	≤0.5	8	≤1	≤1	≤20
37343	≤2	**≥8**	≤0.12	≤1	≤1	≤0.5	8	≤1	≤1	≤20
37761	≤2	**≥8**	≤0.12	≤1	≤1	≤0.5	8	≤1	≤1	≤20
36984	≤2	**≥8**	≤0.12	≤1	≤1	≤0.5	8	≤1	≤1	≤20
37297	≤2	**≥8**	≤0.12	≤1	≤1	≤0.5	8	≤1	≤1	≤20

Abbreviations: AN = amikacin; CEF = ceftiofur; ENR = enrofloxacin; IMI = imipenem; GM = gentamicin; MAR = marbofloxacin; PIP = piperacilline; SXT = trimethoprim-sulfamethoxazole; TET = tetracycline; TOB = tobramycin; bold: resistant according to CLSI breakpoints, ceftiofur* MIC for ceftiofur were interpreted as proposed by Klotz et al. 2017 [18] according to breakpoints for 3^rd^ generation cephalosporines set by CLSI for *Acinetobacter* spp.

## Discussion

Here we provide evidence for the current, critical hygiene challenges faced by horse clinics, most notably mirrored by the detection rate of 10.7% of enteral colonization with ESBL-E, including 10.1% ESBL-producing *E*. *coli* for horses admitted for two frequent reasons, wound infection and signs of colic. Furthermore, we found an overall detection rate of 2.6% ESBL-E and 1.5% *A*. *baumannii* in nasal swabs of equine patients (Table 1). Detection of ESBL-E in the anterior nostrils of horses might represent prior (environmental) contamination, especially in cases of colic, where nasogastric intubation is a standard procedure.

An alarming number of MDR was observed, where 92.7% of the ESBL-producing *E*. *coli* showed phenotypical resistance to at least three classes of antibiotics (Fig 1). This could pose severe therapeutic challenges in case of infectious diseases associated with these isolates [25], especially when considering the antimicrobials legally available for treatment of infectious diseases in horses in Europe (www.ema.europa.eu/ema/; www.hma.eu/).

Caution is advisable when comparing study results with respect to individual settings, study population characteristics, and methodical protocols used for microbiological detection of MDR bacterial species. Here, for example, we chose to avoid the use of antibiotic supplemented enrichment broths to lower the risk of horizontal resistance transfer during overnight selective co-cultivation. Moreover, we studied two groups of horses admitted to the hospital, both representing frequently occurring reasons for equine hospital stay. These groups were selected because they represent a large fraction of admitted animal patients per year. Despite being representative of frequent patient groups admitted to the clinic, the results recorded for these animal patient groups did not necessarily represent all horses with an inpatient stay (n = 1,1134 in 2014 and n = 1,265 in 2015) admitted to the horse clinic.

A study reporting on feacal samples (n = 650) obtained from equine patients attended by different veterinary practices (n = 65) in the United Kingdom using a direct inoculation method reported an ESBL-producing *E*. *coli* rate of 6.3%, with a 95% confidence interval (CI) of 4.1–9.6% [1]. Interestingly, spatial differences were observed concerning detection rates of multidrug resistant *E*. *coli* in horse faeces [1]. Moreover, another study reported 84% (76 of 91) positive ESBL-*E*. *coli* feacal samples obtained from horses, including residents and hospitalized equine patients in a large animal clinical setting [26]. In contrast, a recent study in feral horses on a Canadian island revealed a low ESBL-*E*.*coli* (CTX-M-1) carriage rate (1/508) and 2.7% resistance to tetracycline for horses in non-urban and less populated areas [27].

Although horses included in the study by Apostolakos *et al*. (2017) are not directly comparable with the study population reported here, interesting similarities are apparent, including the predominating ESBL-genes, *bla*_CTX-M-1_ (57%) [26]. An additional study by Dolejska *et al*. reported ESBL-*E*. *coli* harboring the *bla*_CTX-M-1_ gene on IncN and IncI1 conjugative plasmids isolated from horses, staff and environmental smears [28]. Our study and the study of Apostolakos *et al*. (2017) identified these and other typical ESBL plasmid types, such as IncHI1 and IncFIA/B and IncFII [26].

For the *E*. *coli* isolates, MLST typing revealed 22 different sequence types, including eight novel STs. Some of these have previously been reported as STs associated with ESBL-producers, including isolates of human and animal origin such as ST224, ST1586 and ST539 [26, 29, 30] Others have been found in wild birds, including ST1683 (STC10) and ST1730 [31, 32]. With regard to the phylogenetic background, 13/41 equine ESBL-*E*. *coli* were assigned to the ST complex (STC) founded by ST1250, either as ST1250 or single locus variants (slv). Moreover, 8/41 were assigned to STC10.

Interestingly, these finding are in concordance with the data of Apostolakos *et al*. (2017), who reported STC1250 and STC10 among the dominating backgrounds for the ESBL-*E*. *coli* from enterally colonized horses. However, STC10 is the dominant commensal sequence type of *E*. *coli* and these results may therefore reflect its common occurrence and genomic plasticity with respect to niche adaption [33]. More research with respect to niche adaptation of these lineages to the enteral tract of horses is clearly needed.

The possible nosocomial spread of ESBL-producing and multi-drug resistant *E*. *coli* isolates belonging to STC10 CTX-M-1 was reported for hospitalized horses, substantiating the clinical importance of this particular lineage for severe infections in horses [3]. Moreover, an ESBL-*E*. *coli* belonging to ST224 SHV-12 was previously reported from environmental sources (floor, drop down box) [3].

AST results for the nine *A*. *baumannii* reported here (Table 2) revealed that carbapenemase production was absent, although a recent report highlighted the importance of those phenotypes for veterinary clinics, especially for dogs and cats [34].

Since the aim of this study was to determine the current Gram-negative MDR introduction rate into the horse clinic, epidemiological aspects associated with MDR acquisition or origins were not recorded, limiting the results with respect to epidemiological value for e.g. risk factor analysis.

Potential sources of ESBL transmission and subsequent colonization of humans has been investigated extensively during the last few years, including livestock production. A Dutch study published in 2013 rejected the hypothesis that individuals in areas with high broiler densities are at greater risk for ESBL colonization. However, horse ownership was found to increase the risk (OR = 4.69; p ≤0.0001) [35]. Hoek *et al*. (2015) investigated the prevalence and risk factors for faecal carriage of ESBL-producing Enterobacteriaceae in humans living in areas with high or low broiler density in the Netherlands. Interestingly, *E*. *coli* belonging to STC10 were among the most common genotypes identified, and *bla*_CTX-M-1_ genes were more often found compared to other ESBL encoding genes among persons owning or in contact with a horse compared to persons not frequently exposed to horses (p = 0.04) [36].

## Conclusion

Here, we provide evidence for a high rate of introduction of transferable MDR pathogens into a large clinical setting for equine patients, clearly depicting the current challenges for veterinary infection control and work place safety for veterinary staff. Consequently, research is warranted with respect to original sources of ESBL-producing *E*. *coli* in equine medicine. Considering the present situation in veterinary medicine [3, 37, 38], it seems clear that more emphasis on prevention is needed for the safety of veterinary patients and staff and the broader community [39], in particular with regard to the general zoonotic transferability of MDR pathogens causing infections in horses [3, 40].

## Supporting information

S1 Table Whole genome sequence data analysis for ESBL-producing *E. coli* with including transferable resistance genes. WGS data analysis for ESBL-producing *E*. *coli* with including transferable resistance genes (ResFinder 2.1 [21], threshold: 98% ID, 90% minimum length) and plasmids (PlasmidFinder 1.3[22], threshold: 95% ID).
(XLSX)
